# Overweight/obese status associates with favorable outcome in patients with metastatic nasopharyngeal carcinoma: a 10-year retrospective study

**DOI:** 10.1186/s40880-016-0139-6

**Published:** 2016-08-09

**Authors:** Wang Li, Lu-Jun Shen, Tao Chen, Xu-Qi Sun, Ying Zhang, Ming Wu, Wan-Hong Shu, Chen Chen, Chang-Chuan Pan, Yun-Fei Xia, Pei-Hong Wu

**Affiliations:** 1 State Key Laboratory of Oncology in South China, Collaborative Innovation Center for Cancer Medicine, Sun Yat-sen University Cancer Center, Guangzhou, 510060 Guangdong P. R. China; 2 Department of Medical Imaging and Interventional Radiology, Sun Yat-sen University Cancer Center, Guangzhou, 510060 Guangdong P. R. China; 3 Zhongshan School of Medical, Sun Yat-sen University, Guangzhou, 510080 Guangdong P. R. China; 4 Department of Radiation Oncology, Sun Yat-sen University Cancer Center, Guangzhou, 510060 Guangdong P. R. China; 5 Department of Medical Oncology, Sichuan Cancer Hospital and Institute, Second People’s Hospital of Sichuan Province, Chengdu, 610041 Sichuan P. R. China

**Keywords:** Nasopharyngeal carcinoma, Body mass index, Metastasis, Prognosis

## Abstract

**Background:**

Although the prognostic impact of body mass index (BMI) in patients with non-metastatic nasopharyngeal carcinoma (NPC) had been extensively studied, its effect among metastatic NPC patients remains unknown. The purpose of this study was to evaluate the prognostic effect of BMI in patients with metastatic NPC.

**Methods:**

We retrospectively studied 819 patients who were diagnosed with distant metastasis from NPC and received treatment between 1998 and 2007. The patients were divided into three subgroups according to the World Health Organization classifications for Asian populations: underweight (BMI <18.5 kg/m^2^), normal weight (BMI 18.5–22.9 kg/m^2^), and overweight/obese (BMI ≥23.0 kg/m^2^). The associations of BMI with overall survival (OS) and progression-free survival (PFS) were determined by Cox regression analysis.

**Results:**

Of the 819 patients, 168 (20.5%) were underweight, 431 (52.6%) were normal weight, and 220 (26.9%) were overweight/obese. Multivariate analysis adjusted for covariates showed that overweight/obese patients had a longer OS than underweight patients [hazard ratio (HR), 0.64; 95% confidence interval (CI), 0.49–0.84] and normal weight patients (HR, 0.72; 95% CI, 0.57–0.90); no significant difference in PFS was observed among these three groups (*P* = 0.407). Moreover, in stratified analysis, no statistically significant differences in the effect of overweight/obese status among different subgroups were observed.

**Conclusion:**

For patients with metastatic NPC, overweight/obese status was associated with longer OS but not longer PFS compared with underweight or normal weight status.

## Background

Nasopharyngeal carcinoma (NPC) is an endemic head and neck epithelial malignancy, with the highest incidence rate in Southeast Asia [1–3]. Although improvements in radiation techniques and chemotherapy regimens have led to more effective treatments for NPC, 15%–42% of patients still have treatment failures due to distant metastases [4–7]. Once metastasis is diagnosed, the prognosis of patients who are receiving palliative chemotherapy is very poor [8, 9]. Identifying prognostic factors for these patients may lead to the development of new clinical interventions to improve survival.

Body mass index (BMI) is a simple weight-for-height calculation that is commonly used to evaluate the nutritional status in adults. Approximately 10% of patients with non-metastatic NPC are underweight at diagnosis; in terms of probability of recurrence, metastasis, and overall mortality, they have worse outcomes than non-underweight patients [10, 11]. On the other hand, higher BMI seems to be associated with favorable prognosis. In a recent published study of NPC patients, Shen et al. [11] found that the hazard ratio (HR) for death was 0.66 [95% confidence interval (CI) 0.48–0.90] for overweight patients and 0.47 (95% CI 0.23–0.97) for obese patients compared with the baseline of normal weight or underweight patients. Although many studies have been conducted in patients with non-metastatic NPC, none has specifically evaluated the prognostic effect of BMI in patients with metastatic NPC. Long-term palliative chemotherapy can greatly impair the nutritional status of patients with metastatic NPC; therefore, understanding the prognostic effect of BMI may lead to better treatment planning.

In the present study, we investigated the prognostic effects of BMI in patients with metastatic NPC who received systemic chemotherapy. Our previous study showed that NPC patients with different metastatic sites and different numbers of lesions appeared to be a very heterogeneous group in terms of survival [12]. Thus, we additionally performed a subgroup analysis to test the consistency of the effect of BMI.

## Patients and methods

### Patients

We reviewed the medical records of 1005 NPC patients with distant metastasis who were treated at Sun Yat-sen University Cancer Center between January 1998 and December 2007. The inclusion criteria included (1) histological or radiological confirmation of distant metastatic lesion(s); (2) Eastern Cooperative Oncology Group performance status of grade 2 or lower; and (3) received at least one cycle of cisplatin-based chemotherapy as first-line treatment. Exclusion criteria were either of the following: (1) Missing weight measurement at baseline and (2) younger than 18 years. The Hospital Ethics Committee of Sun Yat-sen University Cancer Center approved this study.

### Definition

Primary diseases were staged according to the Union of International Cancer Control (UICC) staging system (6th edition) [13]. To define whether patients presented with distant metastasis when first diagnosed with NPC, metastasis onset was categorized as synchronous or metachronous. The metastatic site, number of metastatic organs, and number of metastases referred to the extent of disease at the time of diagnosis.

Patients’ baseline body weight was measured within 14 days of the start of treatment after metastasis was diagnosed. BMI (kg/m^2^) was categorized according to the World Health Organization recommendations for Asian populations [14]. Because the number of obese patients was relatively few (20 patients), we merged overweight and obese patients and obtained three BMI subgroups: underweight (<18.5 kg/m^2^), normal weight (18.5–22.9 kg/m^2^), and overweight/obese (≥23.0 kg/m^2^).

### Treatment

Most patients who presented with synchronous metastasis at initial NPC diagnosis received a cisplatin plus 5-fluorouracil (5-FU) chemotherapy regimen (known as the PF regimen) before concurrent chemoradiotherapy. The concurrent chemotherapy regimen was either 5-FU plus cisplatin or cisplatin alone. The 5-FU plus cisplatin regimen was 70–100 mg/m^2^ of cisplatin on day 1 plus 500–750 mg/m^2^ of 5-FU from day 2 to day 5 every 3–4 weeks, for 2–3 cycles; the cisplatin regimen was 30–40 mg/m^2^ of cisplatin every week, for 4–6 cycles. For patients who had metachronous metastasis after primary treatment, the first-line regimen was almost exclusively platinum-based—cisplatin in combination with 4–6 cycles of one or two of the following drugs: 5-FU, paclitaxel, gemcitabine, and bleomycin. The patients with progression underwent more than one-line chemotherapy regimen. Treatment discontinuation occurred at patient request or for unacceptable drug toxicity. Local therapies such as surgery, radiotherapy, interventional embolization, and radiofrequency ablation were available for those patients who still had metastatic lesions after chemotherapy.

### Follow-up and endpoints

During palliative chemotherapy, patients were evaluated by computed tomography or magnetic resonance imaging for response every two cycles and then every 3 months until death or the last follow-up (June 30, 2014). The primary outcomes were overall survival (OS) and progression-free survival (PFS). OS was defined as time from the diagnosis of distant metastasis to death by any cause. PFS was defined as time from the diagnosis of distant metastasis to tumor progression or death by cancer.

### Statistical analyses

The Pearson χ^2^ test was used to compare the categorical variables among groups respectively. OS and PFS rates were estimated using the Kaplan–Meier method and compared among the BMI subgroups by the log-rank test. The multiple-adjusted Cox model was used to determine the effect of BMI on survival; covariates included age, sex, UICC T category, UICC N category, metastatic onset, lung metastasis (absent versus present), liver metastasis (absent versus present), bone metastasis (absent versus present), single lesion (no versus yes), number of involved sites, and treatment modality. To test the consistency of the favorable effect associated with overweight/obese status, a subgroup analysis using the multiple-adjusted Cox model was further conducted, with all the covariates, except for stratification factor, included. The Cox regression model, including two main effect parameters and their interaction effect parameters, was used to test the interaction effect between BMI and the other covariates. *P* values less than 0.05 were considered statistically significant. Statistical analyses were performed using the SPSS 20.0 software (IBM SPSS Inc., Chicago, IL, USA).

## Results

### Patient characteristics

A total of 819 patients who met the inclusion criteria were included in this study. Table 1 shows the baseline characteristics of the 819 patients with metastatic NPC. The median age was 45 years (range 18–78 years). Of these patients, 772 (94.3%) had undifferentiated non-keratinizing carcinoma, 31 (3.8%) had differentiated non-keratinizing carcinoma, and 16 (1.9%) had other types; 272 (33.2%) had synchronous metastasis, and 547 (66.8%) had metachronous metastasis. Two hundred seventy-four (33.5%) patients had more than one metastatic site. All patients in either the synchronous or the metachronous group received at least one cycle of platinum-based chemotherapy (median, four cycles). Local therapy for metastases was administered to 212 (25.9%) patients. The median follow-up time for patients was 18 months (range 1–120 months).

**Table 1:** Baseline characteristics by BMI level of patients with metastatic nasopharyngeal carcinoma (NPC)

Variable	All	Underweight	Normal weight	Overweight/obese	*P* value^*^
Total	819	168	431	220	
Age (years)					0.043
<45	419 (51.2)	97 (57.7)	223 (51.7)	99 (45.0)	
≥45	400 (48.8)	71 (42.3)	208 (48.3)	121 (55.0)	
Sex					0.688
Men	681 (83.2)	139 (82.7)	355 (82.4)	187 (85.0)	
Women	138 (16.8)	29 (17.3)	76 (17.6)	33 (15.0)	
UICC T category					0.504
T1–2	369 (45.1)	76 (45.2)	201 (46.6)	92 (41.8)	
T3–4	450 (54.9)	92 (54.8)	230 (53.4)	128 (58.2)	
UICC N category					0.221
N0–1	417 (50.9)	89 (53.0)	227 (52.7)	101 (45.9)	
N2–3	402 (49.1)	79 (47.0)	204 (47.3)	119 (54.1)	
Metastasis onset					<0.001
Synchronous	272 (33.2)	42 (25.0)	135 (31.3)	95 (43.2)	
Metachronous	547 (66.8)	126 (75.0)	296 (68.7)	125 (56.8)	
Lung metastasis					0.709
Absent	469 (57.3)	96 (57.1)	242 (56.1)	131 (59.5)	
Present	350 (42.7)	72 (42.9)	189 (43.9)	89 (40.5)	
Liver metastasis					0.201
Absent	574 (70.1)	118 (70.2)	292 (67.7)	164 (74.5)	
Present	245 (29.9)	50 (29.8)	139 (32.3)	56 (25.5)	
Bone metastasis					0.032
Absent	382 (46.6)	67 (39.9)	219 (50.8)	96 (43.6)	
Present	437 (53.4)	101 (60.1)	212 (49.2)	124 (56.4)	
Solitary lesion					0.010
No	701 (85.6)	155 (92.3)	356 (82.6)	190 (86.4)	
Yes	118 (14.4)	13 (7.7)	75 (17.4)	30 (13.6)	
Number of involved sites					0.592
One	545 (66.5)	107 (63.7)	287 (66.6)	151 (68.6)	
Two or more	274 (33.5)	61 (36.3)	144 (33.4)	69 (31.4)	
Treatment modality					0.322
CT	607 (74.1)	132 (78.6)	313 (72.6)	162 (73.6)	
CT + LT	212 (25.9)	36 (21.4)	118 (27.4)	58 (26.4)	

All values are presented as the number of cases followed by percentage in the parentheses

*BMI* body mass index; *UICC* Union of International Cancer Control; *CT* chemotherapy; *LT* local therapy

* *P* values were computed excluding patients without information in the corresponding variable

For all patients, the median BMI was 21.2 kg/m^2^. A total of 168 patients (20.5%) were underweight, 431 (52.6%) were normal weight, and 220 (26.9%) were overweight/obese. The proportion of overweight/obese was higher in patients who had synchronous metastasis than in those who had metachronous metastasis and were higher in patients aged ≥45 years than in patients aged <45 years. Additionally, the proportion of underweight was higher in patients who had bone metastasis or multiple lesions than in patients who did not have. No significant differences were observed in sex, UICC T category, UICC N category, lung metastasis, liver metastasis, number of involved sites, and treatment modality across the BMI subgroups (Table 1).

### BMI and survival

For the 819 patients included in this analysis, 653 (79.7%) progressions and 498 (60.8%) deaths were recorded. The 1-, 3-, and 5-year OS rates for the whole patient population were 81.4%, 33.7% and 16.7%, respectively; the 1-, 3-, and 5-year PFS rates were 47.6%, 16.6% and 7.1%, respectively.

**Fig. 1:**
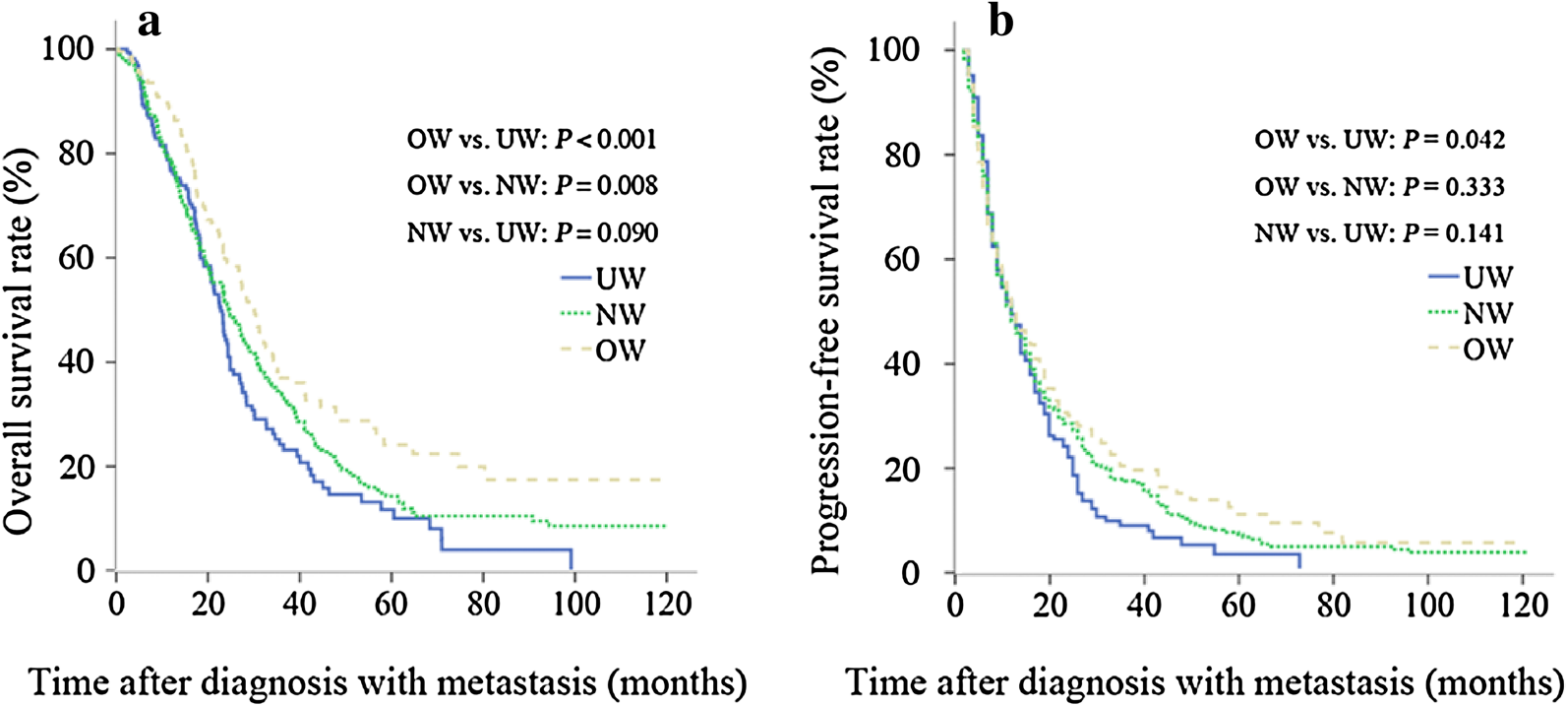
Overall survival (OS) and progression-free survival (PFS) for metastatic nasopharyngeal carcinoma (NPC) patients by body mass index (BMI) levels. Overweight/obese patients had significantly higher OS (**a**) and PFS (**b**) rates than underweight patients, whereas no significant differences were observed in OS and PFS between normal weight and underweight patients. *UW* underweight, *NW* normal weight, *OW* overweight/obese

In univariate analysis, overweight/obese patients had a significantly higher 5-year OS rate than underweight patients (25.9% vs. 12.3%, *P* < 0.001) and normal weight patients (25.9% vs. 14.6%, *P* = 0.008), whereas no significant difference was observed between underweight and normal weight patients (*P* = 0.112; Fig. 1a). In contrast, overweight/obese patients had significantly higher 5-year PFS rates than underweight patients (11.9% vs. 3.5%, *P* = 0.042), whereas no significant differences were observed between normal weight and overweight/obese patients (*P* = 0.333) or between normal weight and underweight patients (*P* = 0.141) (Fig. 1b). Other factors influencing OS and PFS are listed in Tables 2 and 3.

**Table 2:** Univariate and multivariate analyses of covariates associated with overall survival in patients with metastatic NPC

Variable	Univariate		Multivariate	
	HR (95 % CI)	*P*	HR (95 % CI)	*P*
Age (<45 years vs. ≥45 years)	1.23 (1.03–1.46)	0.023	1.28 (1.07–1.53)	0.007
Sex (men vs. women)	1.02 (0.81–1.29)	0.870	1.01 (0.79–1.27)	0.958
UICC T category (T1–2 vs. T3–4)	0.91 (0.77–1.09)	0.310	0.94 (0.78–1.12)	0.480
UICC N category (N0–1 vs. N2–3)	1.33 (1.12–1.59)	0.001	1.39 (1.16–1.67)	<0.001
Metastasis onset (synchronous vs. metachronous)	1.13 (0.94–1.37)	0.199	1.24 (1.01–1.53)	0.039
Solitary lesion (no vs. yes)	0.65 (0.50–0.84)	0.001	0.81 (0.61–1.07)	0.140
Lung metastasis (absent vs. present)	0.90 (0.76–1.08)	0.255	1.04 (0.77–1.41)	0.802
Liver metastasis (absent vs. present)	1.39 (1.16–1.67)	<0.001	1.51 (1.13–2.00)	0.005
Bone metastasis (absent vs. present)	1.20 (1.01–1.44)	0.039	1.31 (1.00–1.73)	0.055
Number of involved sites (one vs. two or more)	1.39 (1.16–1.67)	<0.001	0.97 (0.72–1.29)	0.814
Treatment modality (CT vs. CT + LT)	0.67 (0.55–0.82)	<0.001	0.68 (0.55–0.85)	<0.001
BMI		0.001^a^		0.003^a^
Underweight vs. normal weight	0.84 (0.67–1.04)	0.112	0.90 (0.72–1.12)	0.330
Underweight vs. overweight/obese	0.62 (0.48–0.81)	<0.001	0.64 (0.49–0.84)	0.001

*UICC* Union of International Cancer Control; *CT* chemotherapy; *LT* local therapy; *BMI* body mass index; *HR* hazard ratio; *CI* confidence interval

^a^ *P* value with respect to the significance of differential prognosis between BMI subgroups

**Table 3:** Univariate and multivariate analyses of covariates associated with progression-free survival in patients with metastatic NPC

Variable	Univariate		Multivariate	
	HR (95 % CI)	*P*	HR (95 % CI)	*P*
Age (<45 years vs. ≥45 years)	1.01 (0.87–1.18)	0.863	1.03 (0.88–1.20)	0.760
Sex (men vs. women)	1.04 (0.85–1.27)	0.730	1.04 (0.84–1.27)	0.745
UICC T category (T1–2 vs. T3–4)	0.92 (0.79–1.07)	0.289	0.92 (0.78–1.07)	0.274
UICC N category (N0–1 vs. N2–3)	1.23 (1.05–1.43)	0.009	1.29 (1.10–1.51)	0.002
Metastasis onset (synchronous vs. metachronous)	1.04 (0.88–1.23)	0.647	1.17 (0.98–1.39)	0.091
Solitary lesion (no vs. yes)	0.69 (0.55–0.87)	0.001	0.87 (0.68–1.12)	0.281
Lung metastasis (absent vs. present)	0.87 (0.75–1.02)	0.083	0.98 (0.76–1.27)	0.900
Liver metastasis (absent vs. present)	1.50 (1.27–1.77)	<0.001	1.50 (1.19–1.90)	0.001
Bone metastasis (absent vs. present)	1.27 (1.09–1.48)	0.003	1.33 (1.05–1.68)	0.019
Number of involved sites (one vs. two or more)	1.47 (1.25–1.72)	<0.001	1.08 (0.84–1.38)	0.556
Treatment modality (CT vs. CT + LT)	0.68 (0.57–0.81)	<0.001	0.70 (0.58–0.85)	0.001
BMI		0.139^a^		0.407^a^
Underweight vs. normal weight	0.87 (0.72–1.06)	0.141	0.91 (0.75–1.11)	0.367
Underweight vs. overweight/obese	0.80 (0.64–1.00)	0.042	0.86 (0.68–1.08)	0.181

*UICC* Union of International Cancer Control; *CT* chemotherapy; *LT* local therapy; *BMI* body mass index; *HR* hazard ratio; *CI* confidence interval

^a^ *P* value with respect to the significance of differential prognosis between BMI subgroups

Multiple-adjusted Cox model was used in multivariate analysis with covariates including age, sex, UICC T category, UICC N category, metastatic onset, lung metastasis, liver metastasis, bone metastasis, solitary lesion, number of involved sites, and treatment modality. Collinearity for all the adjusting variables was tested, resulting in variance inflation factors (1.02–2.62) and tolerances (0.44–0.98) within acceptable regression limits. Overweight/obese patients had a significantly lower risk of death compared with underweight patients (HR 0.62; 95% CI 0.48–0.81; Table 2) and normal weight patients (HR 0.72; 95% CI 0.57–0.90), whereas no significant difference was observed in OS between normal weight and underweight patients (HR 0.84; 95% CI 0.67–1.04). The other significant prognostic factors for OS included age, UICC N category, metastasis onset, liver metastasis, and treatment modality. In multiple-adjusted analysis for PFS, BMI was not significant; the significant prognostic factors included UICC N category, liver metastasis, bone metastasis, and treatment modality (Table 3).

To examine the consistency of the effect of BMI in patients with metastatic NPC, we conducted further stratified analysis using the multiple-adjusted model (Table 4). No statistically significant differences were observed in the effects of overweight/obese status on other explanatory variables, but the effect seemed more pronounced in NPC patients with bone metastasis and in patients receiving chemotherapy only. The magnitude of favorable effect of overweight/obese status was similar across the age, sex, UICC T category, UICC N category, and metastasis onset categories.

**Table 4:** Multiple-adjusted HRs for OS by baseline BMI level, stratified by covariates

Stratification covariate	No. of patients	Underweight (HR^a^)	Normal weight [HR (95% CI)^a^]	Overweight/obese [HR (95% CI)^a^]	*P* ^b^
Age (years)					0.675
<45	419	1.0	1.01 (0.74–1.38)	0.74 (0.50–1.09)	
≥45	400	1.0	0.76 (0.55–1.06)	0.54 (0.37–0.79)	
Sex					0.680
Men	681	1.0	0.82 (0.64–1.04)	0.59 (0.44–0.79)	
Women	138	1.0	1.29 (0.67–2.45)	0.93 (0.45–1.94)	
UICC T category					0.471
T1–2	369	1.0	0.84 (0.60–1.17)	0.78 (0.52–1.17)	
T3–4	450	1.0	0.88 (0.65–1.19)	0.49 (0.34–0.70)	
UICC N category					0.501
N0–1	417	1.0	0.83 (0.61–1.14)	0.55 (0.37–0.82)	
N2–3	402	1.0	0.86 (0.60–1.18)	0.67 (0.46–0.98)	
Metastasis onset					0.551
Synchronous	272	1.0	0.94 (0.60–1.49)	0.55 (0.33–0.90)	
Metachronous	547	1.0	0.80 (0.62–1.04)	0.67 (0.48–0.92)	
Lung metastasis					0.761
Absent	469	1.0	0.92 (0.69–1.24)	0.66 (0.46–0.95)	
Present	350	1.0	0.78 (0.54–1.12)	0.62 (0.40–0.94)	
Liver metastasis					0.098
Absent	574	1.0	0.70 (0.54–0.91)	0.45 (0.32–0.62)	
Present	245	1.0	1.39 (0.22–2.19)	1.46 (0.88–2.43)	
Bone metastasis					0.051
Absent	382	1.0	0.83 (0.58–1.18)	0.76 (0.50–1.14)	
Present	437	1.0	0.91 (0.68–1.23)	0.57 (0.39–0.82)	
Solitary lesion					0.062
No	701	1.0	0.90 (0.72–1.14)	0.60 (0.45–0.80)	
Yes	118	1.0	0.35 (0.14–0.86)	0.44 (0.17–1.13)	
Number of involved sites					0.331
One	545	1.0	0.72 (0.57–0.99)	0.53 (0.38–0.74)	
Two or more	274	1.0	1.11 (0.75–1.66)	0.84 (0.52–1.33)	
Treatment modality					0.055
CT	607	1.0	0.92 (0.71–1.19)	0.56 (0.41–0.77)	
CT + LT	212	1.0	0.74 (0.46–1.21)	0.89 (0.52–1.52)	

*OS* overall survival; *BMI* body mass index; *UICC* Union of International Cancer Control; *CT* chemotherapy; *LT* local therapy; *HR* hazard ratio; *CI* confidence interval

^a^Adjusted for age, sex, UICC T category, UICC N category, onset of metastasis, solitary lesion, lung metastasis, liver metastasis, bone metastasis, number of involved sites, and treatment modality, excluding stratification covariate

^b^ *P* for interaction

## Discussion

In the present study, we found that, in patients with metastatic NPC, BMI was an independent prognostic factor for OS but not for PFS. Patients who were overweight/obese had a significantly lower risk of death than underweight and normal weight patients; this effect was more pronounced in patients with bone metastasis and in those who received chemotherapy only.

Several studies reported on the prognostic effect of BMI in patients with NPC [6, 10, 15], and there is increasing awareness that this effect may vary in different subgroups. Huang et al. [16] retrospectively analyzed the data of 400 patients with stage III or stage IVa NPC who received chemoradiotherapy. They found that, compared with normal weight patients, overweight patients had a more favorable OS (HR 0.57; 95% CI 0.39–0.85) and distant failure-free survival (HR 0.61; 95% CI 0.40–0.92) [16]. This finding suggests that nutritional status might also play a critical role in advanced-stage or even end-stage NPC patients. A recent study by Pan et al. [17] evaluated the prognostic effect of anatomic features of metastases in patients with metastatic NPC. They showed that, in the synchronous metastatic group, underweight status was an unfavorable prognostic factor, whereas in the metachronous group, its effect failed to reach significance [17]. However, in their study, covariates associated with the treatment modality were not included in the analysis, and BMI level was simply dichotomized (<18.5  vs. ≥18.5 kg/m^2^), which impeded further interpretation. In our study, we observed a markedly higher OS rate (but not a higher PFS rate) in overweight/obese patients compared with underweight or normal weight patients. Since we observed a differential distribution for age, metastasis onset, bone metastasis, and the number of lesions in the BMI subgroups, we conducted a multiple-adjusted analysis and found that the BMI level was still significant in predicting OS, with its effect consistent in both the synchronous metastasis and the metachronous metastasis groups. In aggregate, these results suggest that BMI level is an important prognostic factor in patients with metastatic NPC.

The reason for the prognostic effect of BMI in patients with metastatic NPC may mainly involve malnutrition. Underweight head and neck cancer patients are more susceptible to malnutrition and even cachexia—both known to be associated with compromised immunity [18, 19], reduced tolerance to oncologic therapies [20, 21], and poor treatment outcome [22–25]—than patients with higher BMI. By contrast, patients who are overweight or obese may tolerate therapy better and therefore may have better outcomes. In addition, comorbidities like diabetes, chronic obstructive pulmonary disease, and cardiovascular diseases could worsen the prognosis of NPC patients; these comorbidities might also lead to low BMI [2, 26]. A recent study of 1001 newly diagnosed NPC patients showed that, before radiation treatment, 15.5% of elderly patients (>70 years old) presented with moderate to severe comorbidities (Adult Comorbidity Evaluation-27 score >2); these patients had lower OS rate (HR 2.63; 95% CI 1.45–4.76) than patients who had no comorbidities or mild comorbidities. However, in our population, the proportion of elderly patients was rather small (16 patients; 1.9%); thus, this may not be an important consideration [27].

We found that pre-treatment BMI level was independently associated with OS in patients with metastatic NPC, but it is interesting that BMI did not have an effect on PFS. Currently, no curative therapy is available for metastatic NPC. One plausible explanation for this may be that, for patients with metastatic NPC, being overweight or obese does not guarantee that any given oncologic therapy will have improved efficacy; being overweight or obese may, however, associate with a higher tolerance to continuous treatment, resulting in superior long-term benefits [9, 28]. Moreover, this may be partly supported by our preliminary data that the overweight/obese group received more cycles of palliative chemotherapy after metastasis diagnosis than the other two groups (overweight/obese: median, five cycles; normal weight: median, three cycles; underweight: median, four cycles).

Our study showed the clinical course of a large cohort of patients with metastatic NPC. OS varied among the patients (range 1–120 months), and the 5-year OS rates after metastasis for overweight/obese, normal weight, and underweight patients were 25.9%, 14.6% and 12.3%, respectively. These results indicate that long-term survival is possible for some patients and that more aggressive multimodality treatment should be encouraged for patients with high baseline BMI. Interestingly, the unfavorable prognostic effect of underweight status in patients who received chemotherapy combined with local therapy was absent; this suggests that patients with localized metastatic lesions who receive local therapy might overcome the negative effect of low baseline BMI. In addition, since patients with higher BMI levels may be more tolerant to intensive treatment, and since a higher dosage or denser interval of chemoradiotherapy could potentially be beneficial, future clinical trials that investigate the optimal dosage and interval of chemotherapy are important and warranted.

Our results suggest that, for patients with metastatic NPC, certain interventions that target underweight status or malnutrition might be beneficial. For the past decade, ample evidence has shown that, for patients with head and neck cancer, adequate nutrition support before and during treatment can decrease the severity of adverse effects, minimize weight loss, and improve outcomes [29, 30]. Studies have suggested that patients with head and neck cancer who have a BMI less than 20 kg/m^2^ should receive further assessment, intensive counselling, and nutrition support [31–33]. However, owing to a lack of high-quality studies, recent reviews were not able to provide evidence for or against a role of nutrition support in head neck cancer patients [31, 34, 35]. Additionally, no study has specifically focused on the effect of nutrition intervention on patients with NPC. Therefore, multicenter randomized controlled trials and well-designed observation studies that examine nutrition intervention are needed.

This study had several limitations. First, it was a retrospective study. Second, BMI was measured only at the diagnosis of metastasis, and further changes were not considered owing to the difficulty of assessing changes influenced by adverse effects of different chemotherapy/local therapy. Finally, the applied modes of chemotherapy and local therapy varied, which might have a confounding effect. For these reasons, our findings need to be validated in a multi-institutional prospective study.

## Conclusions

This study showed that, for patients receiving palliative chemotherapy, overweight or obese at baseline was associated with longer OS compared with underweight or normal weight. This association was similar across patient subgroups and seemed pronounced in patients with bone metastasis and in those who received chemotherapy only. These data emphasize the link between malnutrition and the survival of patients with metastatic NPC and suggest that nutritional intervention may be recommended for underweight and normal weight patients.

## Abbreviations

NPC: nasopharyngeal carcinoma
BMI: body mass index
PFS: progression-free survival
OS: overall survival
HR: hazard ratio
CI: confidence interval
